# Unveiling the Molecular Mechanisms of Browning in *Camellia hainanica* Callus through Transcriptomic and Metabolomic Analysis

**DOI:** 10.3390/ijms252011021

**Published:** 2024-10-14

**Authors:** Kunlin Wu, Yanju Liu, Yufen Xu, Zhaoyan Yu, Qiulin Cao, Han Gong, Yaodong Yang, Jianqiu Ye, Xiaocheng Jia

**Affiliations:** 1 Hainan Key Laboratory of Tropical Oil Crops Biology, Coconut Research Institute, Chinese Academy of Tropical Agricultural Sciences, Wenchang 571339, China; wkk1225@163.com (K.W.);; 2 Institute of Scientific and Technical Information, Chinese Academy of Tropical Agricultural Sciences, Haikou 571101, China

**Keywords:** *Camellia hainanica*, metabolomic, transcriptomic, flavonoid, gene expression

## Abstract

*Camellia hainanica* is one of the camellia plants distributed in tropical regions, and its regeneration system and genetic transformation are affected by callus browning. However, the underlying mechanism of *Camellia hainanica* callus browning formation remains largely unknown. To investigate the metabolic basis and molecular mechanism of the callus browning of *Camellia hainanica*, histological staining, high-throughput metabolomics, and transcriptomic assays were performed on calli with different browning degrees (T1, T2, and T3). The results of histological staining revealed that the brown callus cells had obvious lignification and accumulation of polyphenols. Widely targeted metabolomics revealed 1190 differentially accumulated metabolites (DAMs), with 53 DAMs annotated as phenylpropanoids and flavonoids. Comparative transcriptomics revealed differentially expressed genes (DEGs) of the T2 vs. T1 associated with the biosynthesis and regulation of flavonoids and transcription factors in *Camellia hainanica*. Among them, forty-four enzyme genes associated with flavonoid biosynthesis were identified, including *phenylalaninase* (*PAL*), *4-coumaroyl CoA ligase* (*4CL*), *naringenin* via *flavanone 3-hydroxylase* (*F3H*), *flavonol synthase* (*FLS*), *Chalcone synthase* (*CHS*), *Chalcone isomerase* (*CHI*), *hydroxycinnamoyl-CoA shikimate transferase* (*HCT*), *Dihydroflavonol reductase* (*DFR*), *anthocyanin reductase* (*LAR*), *anthocyanin synthetase* (*ANS*), and *anthocyanin reductase* (*ANR*). Related transcription factors *R2R3-MYB*, *basic helix-loop-helix* (*bHLH*), and *WRKY* genes also presented different expression patterns in T2 vs. T1. These results indicate that the browning of calli in *Camellia hainanica* is regulated at both the transcriptional and metabolic levels. The oxidation of flavonoids and the regulation of related structural genes and transcription factors are crucial decisive factors. This study preliminarily revealed the molecular mechanism of the browning of the callus of Camellia hainanensis, and the results can provide a reference for the anti-browning culture of *Camellia hainanica* callus.

## 1. Introduction

*Camellia oleifera* belongs to the largest genus of Theaceae, and along with olive, oil palm, and coconut, they are known as the world’s four major woody oilseed crops [1]. Oil tea camellia has been cultivated in China for more than 2300 years, with a national planting area of 4.7 million hectares and an annual output of 800,000 tons of camellia tea seed oil nowadays. *Camellia hainanica* is one of the camellia species that grows in tropical regions and is a special ecological type or varietas of Camellia drupifera. Owing to the unique geographical location and favorable climatic conditions of Hainan Island, the tea seed oil has a unique flavor and higher quality than mainland China strains [2]. Therefore, tea seed oil originated in Hainan is widely used in food, medicine, cosmetics, and other fields [3]. With the rapid development of biotechnology, tissue culture technology has become an important means of rapid propagation and genetic improvement of *Camellia hainanica*.

In plant tissue culture, the phenomenon of explant and callus browning is widespread, which means that the surface or inside of the explant and callus is brown. The occurrence of browning not only affects the normal growth and development of explants but also leads to death, which is an urgent problem to be solved in plant tissue culture [4]. Although explants such as cotyledons and hypocotyls of *Camellia hainanica* can easily produce calli, browning of calli is common in the process of tissue culture. Callus browning retards callus growth, inhibits cell regeneration, leads to early senescence and even cell death, and severely hinders plant tissue culture [5]. In fact, many transgenic plants are difficult to regenerate due in large part to the problem of rapid and frequent callus browning in plants [6]. This reduces the growth quality and differentiation ability of calluses and limits their application in production.

In recent years, many scholars have conducted extensive research on the phenomenon of browning. These researchers believe that browning can be divided into enzymatic and non-enzymatic types. The non-enzymatic type of browning is mainly affected by the adverse external environment, resulting in cell death. Enzymatic browning is mainly caused by the mixture of phenol compounds and polyphenol oxidase in explants or calli and the reaction with oxygen to produce quinones [7]. As respiratory transmitters, phenolic substances participate in respiratory metabolism and are in dynamic equilibrium with the redox of quinones [8]. In normal plant tissues, polyphenol substances are distributed in the vacuoles of cells, and enzymes are distributed in various plastids and cytoplasm. This regional distribution prevents contact between enzymes and polyphenols, thus avoiding the occurrence of enzymatic browning in normal tissues [9]. When plant tissue cells are damaged, phenolic substances come into contact with oxygen and oxidase. They are rapidly oxidized to O-quinone, which is then formed by non-enzymatic polymerization to form brown pigment or melanin [10].

Non-enzymatic browning may occur in plant tissue culture, but enzymatic browning causes most of it. So, the degree of callus browning largely depends on the content of phenolic substances and the degree of oxidation. Phenolic compounds are secondary metabolites of plants, and most of them are synthesized through the flavonoid biosynthetic pathway. Phenylpropane compounds include phenylpropane esters, lignin, flavonoids, and hydroxycinnamic acid amides [11,12]. Flavonoids (PFs) exhibit various biological activities, including antimicrobial, anti-inflammatory, and anticancer activities [13]. In addition, flavonoids are considered antioxidants with the potential to scavenge free radicals and quench reactive oxygen and nitrogen species [14].

Phenylalanine is catalyzed by special enzymes to form various phenols, lignin, anthocyanins, alkaloids, and other compounds, and the produced phenols provide substrates for the browning reaction [15,16]. Flavonoids are produced by a branch of the phenylalanine pathway, the first and key enzyme of which is phenylalaninase (PAL) [17]. The product catalyzed by PAL is formed by cinnamic acid 4-hydroxylase (C4H) and 4-coumarol CoA ligase (4CL) to form 4-coumarol CoA [18]. 4-coumaroyl-CoA can be used as the substrate of hydroxycinnamoyl-CoA shikimate transferase (HCT), which causes the formation of coumarin-quinic acid or country-shikimic acid [19]. Alternatively, 4-coumaroyl-CoA forms a chalcone under the action of Chalcone synthase (CHS). The chalcone is catalyzed by Chalcone isomerase (CHI) production of naringin [20]. Next, naringin is catalyzed by flavonoid 3-hydroxylase (F3H) to produce dihydroflavonol and then catalyzed by dihydroflavonol reductase (DFR) to form proanthocyanidins (PAs) [21,22]. PAs, also known as condensed tannins, can be anthocyanin reductase (LAR), anthocyanin synthetase (ANS), and anthocyanin reductase (ANR), which catalyze the generation of anthocyanins [23,24]. In addition to enzymes and corresponding structural genes, PF biosynthesis is also regulated by transcription factors (TFs) [25]. The transcription factors R2R3-MYB, basic helix-loop-helix (bHLH), and WRKY, which are closely related to flavonoid biosynthesis, have been shown to be directly or indirectly involved in regulating flavonoid biosynthesis in many species [26,27].

In summary, exploring the biosynthesis of flavonoids can elucidate the internal mechanism of *Camellia hainanica* callus browning. However, there have been no reports on the dynamic changes in the metabolism and transcription of phenols and flavonoids in *Camellia hainanica* during callus browning. In fact, the molecular mechanism of callus browning has been elucidated in some species, but the molecular mechanism of callus browning in *Camellia hainanica* is still not fully understood. In this study, the calli of *Camellia hainanica* at different stages of browning were stained histologically, as were the analytical result of transcriptomics and metabolomics. They were integrated to study the dynamic changes in metabolite and genetic expression to reveal the molecular mechanism of callus browning in *Camellia hainanica* and provide a new perspective to solve the problem of callus browning. This study provides theoretical support for improving callus culture conditions, tissue culture efficiency, and genetic improvement of *Camellia hainanica*. In the future, we will continue to study the functions of these genes and metabolites, as well as their regulatory role in callus browning, to provide more effective strategies for the tissue culture and genetic improvement of *Camellia hainanica*.

## 2. Results

### 2.1. Observation of Calli with Different Browning Degrees

**Figure 1:**
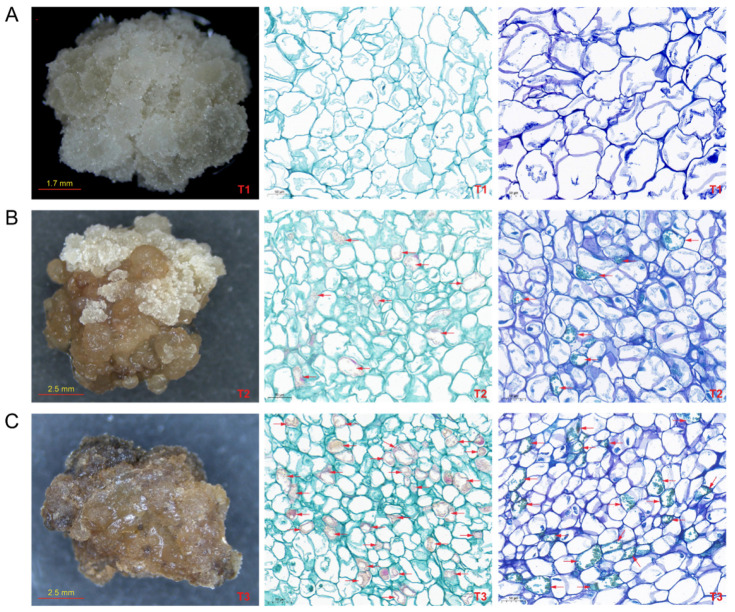
Classification and histological observation of callus browning in *Camellia hainanica*. (**A**) Unbrowning callus (T1) morphology, safranin O-fast green- and toluidine blue-stained sections. (**B**) Browning callus (T2) morphology, safranin O-fast green, and toluidine blue-stained sections. (**C**) Severe browning callus (T3) morphology, safranin O-fast green- and toluidine blue-stained sections. Note: In the stained safranin O-fast green sections, arrows indicate red-stained lignified matter; in the toluidine blue-stained sections, arrows indicate the accumulation of green-colored polyphenolic materials.

In this study, we induced calluses from the leaves of a new high-yield *Camellia hainanica* cultivar, “Reyan No. 2”, as explants. The results revealed that calli could be generated from 15-day-old immature cotyledons. During the first 2 weeks of culture, the newly induced callus initially appeared healthy and white crystalline; this stage represented the T1 callus (Figure 1A). These calli tend to turn browning after 6–8 weeks, and most are light brown in volume, at which point they are T2 calli (Figure 1B). After 14–16 weeks, the whole tissue turned brown and became a T3 callus (Figure 1C), after which it gradually died. We prepared sections of calluses at the T1, T2, and T3 stages. We stained them with saffranine solid green and toluidine blue to evaluate the morphology and viability of the callus cells at the different browning stages. The results revealed that the unbrowning callus (T1) did not accumulate substances in the cells and that the cell wall was green (Figure 1A). In contrast, some brown calli (T2, T3) accumulated red substances in the cells (Figure 1B,C), and the cell wall was red. With increasing browning degree, more cells appeared red, indicating lignin accumulation during callus browning. The red color of these cells is due to the saffranine solid green stain, which stains lignified and cork cells red. After toluidine blue staining, no accumulation of green matter was observed in healthy callus cells. However, green matter accumulation was observed in T2 and T3 callus cells, and T3 callus cells presented a greater accumulation of green matter. In fact, these green accumulations are phenolic compounds stained with toluidine blue. In conclusion, the results of histological staining revealed that the *Camellia hainanica* callus cells were lignified and that phenolic compounds continuously accumulated during the browning process.

### 2.2. Metabolite Profiles of Camellia hainanica Calli at Different Browning Stages

**Figure 2:**
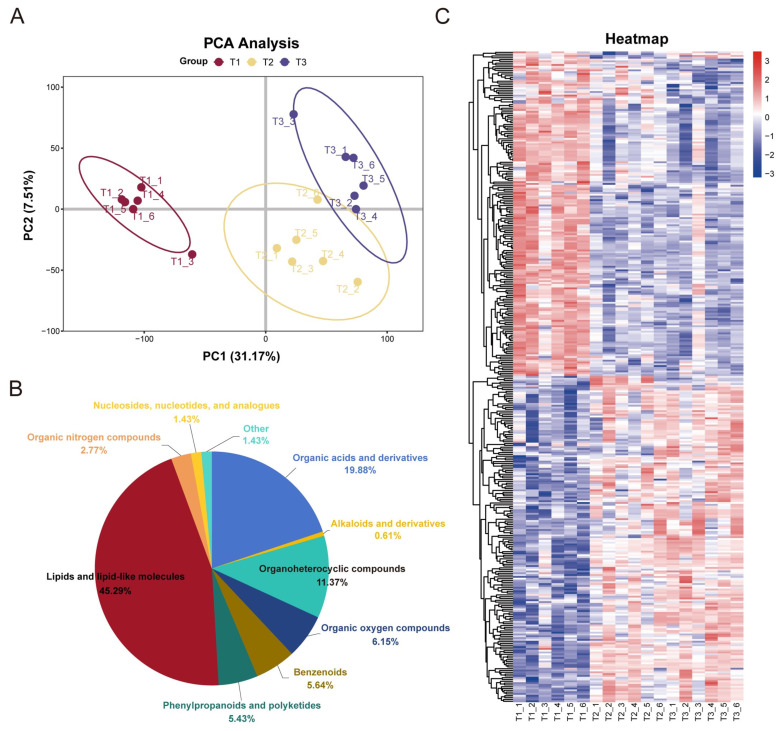
Metabolome profiling in *Camellia hainanica* callus at different browning stages. (**A**) PCA of metabolites in the T1, T2, and T3 phases of the camellia callus; (**B**) Proportions of all metabolic components identified in the callus; (**C**) Cluster heatmaps of all differentially abundant metabolites.

To identify the compounds associated with the browning of calluses, the metabolites of T1, T2, and T3 were determined, and a metabolomic analysis was performed to explore the potential metabolic factors affecting the browning of the *Camellia hainanica* callus. The results of metabolite group PCA revealed that the T1, T2, and T3 callus samples were well separated, indicating that callus browning had a significant effect on the metabolic level and that callus samples at the same browning stage tended to cluster together. Notably, the T1, T2, and T3 samples were clearly separated, suggesting that the difference between unbrowning and browning tissues was more significant (Figure 2A). Metabolomic data revealed that 1109 differentially accumulated metabolites (DAMs) were detected and roughly grouped into ten categories. Among these metabolites, “lipids and lipid-like molecules”, “organic acids and derivatives”, “organoheterocyclic compounds”, and “organic oxygen compounds” accounted for the largest proportions, 45.29%, 19.88%, 11.37%, and 6.15%, respectively. Among them, flavonoid biosynthesis-related phenylpropanoids and polyketides were ranked sixth, accounting for a total of 53 DAMs (Figure 2B). Figure 2C shows clustering heatmaps of all the differentiated metabolites in the metabolome.

### 2.3. Identification of DAMs during Camellia hainanica Callus Browning

**Figure 3:**
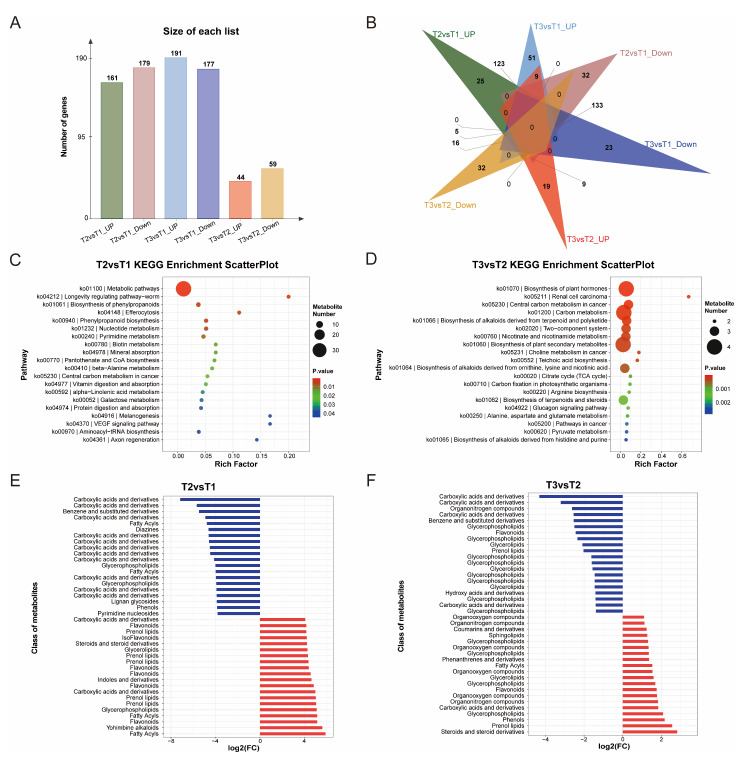
Differential expression of metabolites in *Camellia hainanica* callus. (**A**) Histogram of DAMs up- and downregulated in different groups. (**B**) Venn diagram of the DAMs for different groups. (**C**) KEGG enrichment analysis of DAMs in the T2 vs. T1 comparison. (**D**) KEGG enrichment analysis of DAMs in the T3 vs. T2 comparison. (**E**) Fold changes in the 40 most up- or downregulated DAMs in the T2 vs. T1 comparison. (**F**) Fold changes in the top 20 most up- or downregulated DAMs in the T3 vs. T2 comparison. Note: The most upregulated and downregulated DAMs are shown in red and blue, respectively.

The differential cumulative metabolites (DAMs) with significant differences were screened with *p* < 0.05 VIP > 1 as the index. The results revealed 340 DAMs in T2 vs. T1, of which 161 were upregulated and 179 were downregulated. There were 103 DAMs in T3 vs. T2, of which 44 DAMs were upregulated and 59 DAMs were downregulated. Compared with T1, T3 had a total of 368 DAMs, of which 191 DAMs were upregulated and 177 DAMs were downregulated (Figure 3A). The Venn diagram shows the downregulation of DAMs in the different comparison groups (Figure 3B). In summary, the numbers of metabolites identified by T2 vs. T1 and T3 vs. T1 were similar.

The Kyoto Encyclopedia of Genes and Genomes (KEGG) database was used to analyze the pathways of different metabolites detected in the T1, T2, and T3 stages of the callus of Hainan camelial oil tea to study the influence of different browning degrees on the DAM enrichment pathway of the callus. A total of 1289 metabolites were annotated to 96 metabolic pathways, 33 of which were significantly enriched (*p* ≤ 0.05). The top 20 pathways in the T2 vs. T1 comparison were selected to draw a KEGG bubble map, as shown in Figure 3B. The top six pathways were metabolic pathways, biosynthesis of secondary metabolites, biosynthesis of cofactors, biosynthesis of phenylpropanoids, nucleotide metabolism, and phenylpropanoid biosynthesis. Among them, phenylpropanes and phenylpropanes are abundant in biosynthetic pathways. In T3 vs. T2, DAM enrichment was associated with mainly the biosynthesis of plant hormones, carbon metabolism, the biosynthesis of alkaloids derived from terpenoids and polyketides, two-component systems, nicotinate and nicotinamide metabolism, the biosynthesis of plant secondary metabolites, tea acid biosynthesis, and the biosynthesis of alkaloids derived from the ornithine, lysine, and nicotinic acid pathways (Figure 3C). These pathways are more related to the oxidative stress response of plants, which indicates that from the T2 to T3 stages, oxidative stress increases significantly with increasing browning of the camellia callus.

In addition, the identified DAMs presented significantly different patterns, with 11 of the top 20 downregulated DAMs in the T2 vs. T1 comparison group being organic acids and their derivatives. In contrast, the first 20 upregulated DAMs included 5 flavonoids, 5 prenollipids, and 3 fatty acyl groups (Figure 3E). In the T3 vs. T2 comparison, 8 of the first 20 downregulated DAMs were glycerophospholipids, 5 were carboxylic acids and derivatives, and 3 were glycerolipids. Among the top 20 upregulated DAM compounds, 6 were organooxygen compounds, 4 were glycerophospholipids, and 1 was flavonoid (Figure 3F).

### 2.4. Weighted Gene Co-Expression Network Analysis of Metabolites

**Figure 4:**
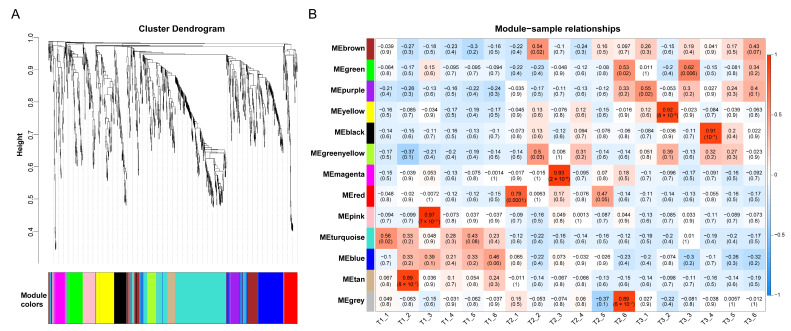
Correlation study of metabolites of *Camellia hainanica* callus via WGCNA. (**A**) Cluster dendrogram of each sample. (**B**) Module–trait relationship heatmap; each cell contains the corresponding correlation and *p* value. The table is color-coded by correlation according to the color legend.

WGCNA (https://www.omicstudio.cn/tool, accessed on 5 August 2024) is a powerful tool for identifying groups of metabolites with highly synergistic variations. Instead of focusing only on differentially abundant metabolites, WGCNA uses information on thousands or tens of thousands of the most varied metabolites or all metabolites to identify metabolite sets of interest and perform significant association analysis with phenotypes. In this study, 480 DAMs identified via WGCNA were clustered into 12 different coexpression modules, and the highly related metabolites were classified into the same module, which could further explore the relationships between metabolites and calli with different browning degrees. Metabolites that do not belong to these modules are shown in grey (Figure 4). The top five genes encoding metabolite coexpression modules are as follows: the MEturquoise module contains 355 DAMs; the MEblue module contains 156 DAMs; the MEbrown module contains 87 DAMs; the MEyellow module contains 83 DAMs; and the MEgreen module contains 70 DAMS. The MEBrown module contains catechins, proanthocyanidins, and other flavonoid-related metabolites. In addition, we associated each coexpression module with calli with different degrees of browning via Pearson correlation coefficient analysis (Figure 4B).

### 2.5. Transcriptome Quality Control

To improve the reliability of the transcriptome experiment, six bioreplicates were performed on the experimental samples in this study, thereby reducing the possibility of random errors during the experiment and providing more accurate genetic data for subsequent analysis. After sequencing quality control, a total of 98.27 GB of clean data were obtained. The average cleaning data of 18 samples reached 5.46 GB. Effective reads were compared to the reference genome of *Camellia hainanica*, and the percentage of all samples was greater than 82.08%. The distribution of the Q20 and Q30 bases in each sample ranged from 92.78% to 97.91%. The GC content ranged from 44.06% to 44.91%, indicating high-quality transcriptome sequencing data (Table 1).

### 2.6. Differential Expression and Enrichment Analysis of Genes

**Figure 5:**
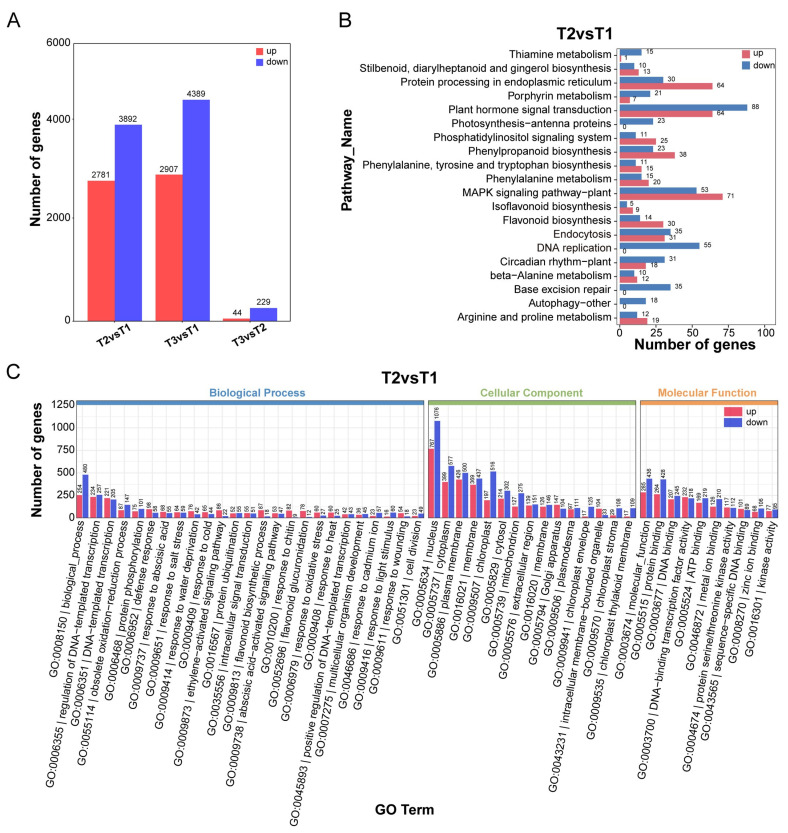
Integrated transcriptomic analysis of *Camellia hainanica* calli at different browning stages. (**A**) Numbers of upregulated and downregulated DEGs for each comparison. (**B**) KEGG enrichment analysis of DEGs in the T2 vs. T1 comparison. (**C**) GO enrichment analysis of DEGs in the T2 vs. T1 comparison.

Subsequently, 18 RNA-seq libraries were screened, and 39,916 genes were identified and annotated. Using |log2FoldChange| as a screening criterion, paired comparisons were conducted with *p* < 0.05 and ≥1 as screening criteria to determine the differentially expressed genes (DEGs) at 3 developmental stages. In the pairwise comparisons of T2 vs. T1, T3 vs. T2, and T3 vs. T1, a total of 6673 (2781 up/3892 down), 273 (44 up/229 down), and 7296 (2907 up/4389 down) genes were identified in T2 vs. T1 (Figure 5A). The results revealed that the browning of calluses was associated with changes in gene expression, and these significantly changed genes may be involved in regulating the browning of calluses.

KEGG pathway enrichment analysis revealed that DEGs in T2 vs. T1 were enriched mostly in biological processes, such as “plant hormone signal transduction”, “plant–pathogen interaction”, “MAPK signaling pathway–plant”, “protein processing in the endoplasmic reticulum”, and “starch and sucrose metabolism” (Figure 5B). The top six pathways enriched with the DEGs from the T3 vs. T2 and T3 vs. T1 comparisons also included “Phenylalanine metabolism” and “Phenylpropanoid biosynthesis”. Consequently, DEGs from the T2 vs. T1 comparison were deemed more tightly associated with *Camellia hainanica* callus browning and were therefore subjected to further analysis. Gene Ontology (GO) enrichment analysis revealed that the DEGs were enriched in three functional categories, namely, molecular function (MF), cell component (CC), and biological process. In the CC category, the DEGs enriched in the nucleus, cytoplasm, plasma membrane, membrane, and chloroplast were the most abundant. In the BP category, DNA-templated transcription, response to chitin, and regulation of DNA-templated transcription were the most abundant. Among MFs, molecular function, DNA-binding transcription factor activity, and protein binding were the most abundant (Figure 5C). In conclusion, the enrichment analysis results revealed that browning activated the stress response of calluses and changed the expression of genes involved in the phenylpropanoid and flavonoid biosynthesis pathways.

### 2.7. Heatmap of the Gene Expression of the Flavonoid Biosynthetic Pathway during Callus Browning

**Figure 6:**
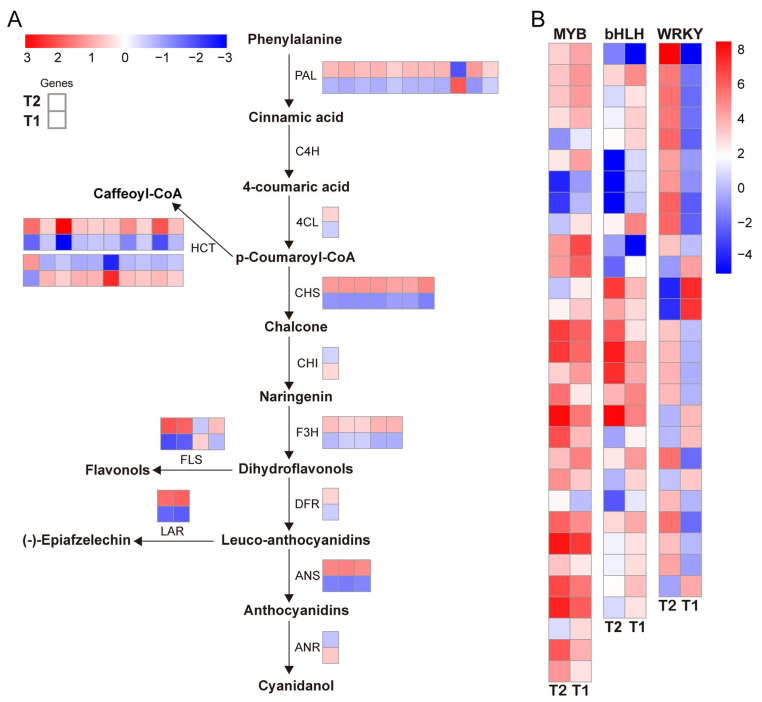
DEGs in the T2 vs. T1 comparison group for reference regulation and flavonoid biosynthesis. (**A**) Schematic representation of the relative expression levels of structural genes in the flavonoid biosynthesis pathway. The two columns of heatmaps from top to bottom represent the T2 and T1 stages, respectively. The lines of the heatmap represent different gene copies, and the diagonal arrows represent multistep enzyme reactions. (**B**) Thermograms of *MYB*, *bHLH*, and *WRKY* transcription factors with differential expression; red and blue indicate significant up–down and down–down, respectively.

Both the metabolome and transcriptome data revealed significant enrichment of metabolites and DEGs in the “flavonoid biosynthesis” pathway in the T2 vs. T1 comparison (Figure 5B). These results suggest that “flavonoid biosynthesis” and the transfer of flavonoids to proanthocyanidins may be key steps in regulating callus browning. To verify the role of the flavonoid biosynthesis pathway in the browning process of *Camellia hainanica* calli, we conducted a comparative expression analysis of all structural genes in this pathway and generated heatmaps. Forty-four DEGs from 11 families were identified in tea callus at the T2 vs. T1 browning stage (Figure 6A). The expression of ten *PAL* genes, one *4CL* gene, seven *CHS* genes, five *F3H* genes, three *flavonol synthase* (*FLS*) genes, two *LAR* genes, and three *ANS* genes in the main flavonoid synthesis pathway was upregulated at the T2 stage; each has one gene. The expression of PAL, CHI, FLS, and ANR was downregulated in the T2 phase. The ANR controlling the biosynthesis of flavan-3-ols on the prebody surface was significantly downregulated, indicating that the biosynthesis of tannins in the camellia callus was conducted mainly through LAR branches. The 20 differentially expressed *HCT* genes identified presented different expression patterns; 11 DEGs were significantly upregulated at the T2 stage, and 9 DEGs were significantly downregulated at T2. In addition, the expression patterns of the transcription factors *MYB*s, *bHLH*, and *WRKY*s, which regulate flavonoid biosynthesis, differed between T2 and T1. Overall, the comparative expression analysis of calli browning at T2 and T1 revealed that most flavonoid biosynthesis genes were significantly upregulated at the T2 browning stage, which may strongly promote the accumulation of flavonoids and provide a large number of substrates for the synthesis of lignin and phenols.

### 2.8. Analysis of Correlations between Genes and Metabolites

**Figure 7:**
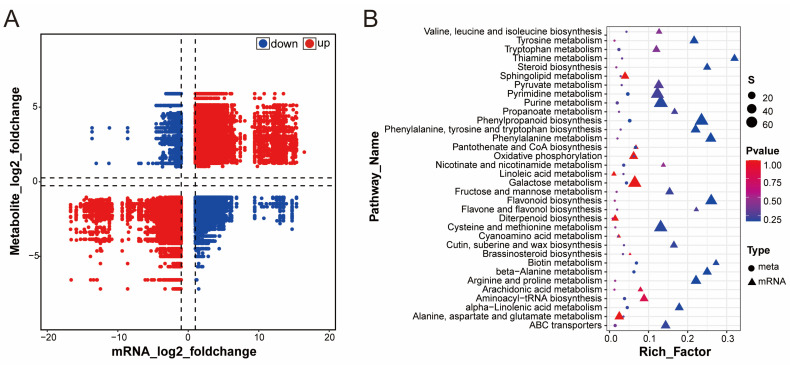
T2 vs. T1 metabolites and gene correlation analysis diagram. (**A**) Nine-quadrant map of metabolites and gene regulation of T2 vs. T1. (**B**) Metabolic pathway map of T2 vs. T1 metabolites and gene regulation.

The correlations between all genes and metabolites were calculated via the Pearson correlation method, and the genes and metabolites with high expression correlations were subsequently screened according to the correlation coefficient and correlation *p*-value. After screening, the expression trends of genes and metabolites were analyzed via a nine-quadrant diagram. As shown in Figure 7, at T2 vs. T1, the expression trends of most genes and metabolites are consistent, indicating that most genes may positively regulate metabolites. In contrast, the expression trends of some genes and metabolites are opposite, and these genes negatively regulate metabolites.

## 3. Discussion

The browning of plant calli is a common problem in tissue culture, severely affecting the callus’s growth and regeneration ability and subsequently affecting the efficiency of plant reproduction and genetic transformation. It is especially common in callus cultures of woody plants and plants rich in secondary metabolites. As a tropical woody oil, *Camellia hainanica* accumulates a large amount of phenolic substances in its organs [28,29]. In plants, the phenolic content increases to resist the effects of harsh environmental conditions, but excessive accumulation of phenolic compounds in the callus leads to increased production of quinones, leading to severe browning [30]. Due to the frequent callus browning, *Camellia hainanica*’s regeneration system and genetic transformation are still challenging. Therefore, studying the mechanism of *Camellia hainanica* callus browning is very important if we want to change the situation of browning easily.

### 3.1. Browning of Camellia hainanica Callus Was Associated with the Accumulation of Phenols in the Cells

Most callus browning phenomena are caused mainly by the accumulation and oxidation of phenolic substances. The browning of calluses caused by the accumulation of phenols affects their morphology and physiological characteristics and may cause cell death and tissue degeneration. In previous studies on tea tissue culture, researchers adjusted the hormone ratio and added antioxidants to prevent the callus from browning during development [31]. In this study, sections of calli with different degrees of browning were stained, and it was found that browning greatly harmed *Camellia hainanica* calli. With increasing browning, lignin and phenolic substances accumulate in the callus cells. These results are similar to those previously reported in several plant species, such as lotus, scots pine, pear, and walnut [15,32,33,34]. Obviously, similar to other plants, the oxidation of phenolic substances may cause the browning of *Camellia hainanica* calli. Therefore, in the tissue culture of *Camellia hainanica*, the methods of inhibiting browning in other plants can be referred to. For example, *Camellia hainanica* tissue with fewer phenolic substances can be selected as explants during callus induction; adsorbents such as activated carbon and polyvinyl pyrrolidone (PVP) were added to the medium for adsorption of phenolic substances [35], or antioxidants such as citric acid and vitamin C were added to reduce the oxidation of phenolic substances [36,37].

### 3.2. Metabolic and Transcriptomic Changes during Callus Browning

Studies on callus browning in other plants have been carried out mainly at the physiological level, that is, to explore the dynamic changes in polyphenol content in calluses and the activity of related enzymes such as polyphenol oxidase (PPO) and peroxidase (POD) [38]. However, our research reveals that many changes occurred in the *Camellia hainanica* callus during the browning process at the metabolic and genetic levels. We comprehensively evaluated the changes in metabolite profiles and gene expression levels in calli T1, T2, and T3 of *Camellia hainanica* with different browning degrees. In metabolomics, a total of 361 different metabolites, such as phenylpropanoids, flavonoids, and organic acids, were identified in these three stages. Among them, there were 340 different metabolites in T2 vs. T1, 161 upregulated metabolites, and 179 downregulated metabolites in T2 compared with T1. In the RNA-Seq test, 39,916 genes were found and annotated in the RNA libraries of the T1, T2, and T3 stages. Using |log2Fold Change| ≥ 1 and corrected *p* < 0.05 as screening conditions, paired comparisons were conducted to determine the DEGs at 3 developmental stages. A total of 6673 T2 vs. T1 DEGs were identified (2781 upregulated, 3892 downregulated).

### 3.3. DEGs of a Large Number of Flavonoid Biosynthetic Pathways Were Significantly Enriched in Brown Calli

Flavonoids are among the main metabolites in plants and have antioxidant, detoxification, and ROS-scavenging activities [14]. This study used the transcriptome and metabolome to identify the structural genes encoding flavonoid biosynthesis in *Camellia hainanica*. A total of 45 DEGs were identified in T2 vs. T1. These genes play specific and critical roles in accumulating specific flavonoids, including *PAL*, *4CL*, *HCT*, *CHI*, *CHS*, *FLS*, *LAR*, *ANS*, *ANR*, etc. PAL is the first rate-limiting enzyme in phenylpropanoid biosynthesis and plays crucial roles in cell differentiation, lignification, flavonoid synthesis, and plant stress [39]. *PAL* gene expression activation is considered to be the main factor leading to the browning of lettuce, lotus, and other plants [40,41]. An increase in *PAL* gene expression and enzyme activity can promote the synthesis of lignin and the phenolic substrate cinnamic acid [42]. Ten of the eleven *PAL* genes identified in this study were upregulated in T2. The upregulation of high levels of *PAL* increased the lignin content and the synthetic substrate of phenolic substances in the brown callus cells of *Camellia hainanica*, which was consistent with the physiological phenomenon of increased accumulation of lignin and phenolic substances observed in the cell sections. HCT is one of the key enzymes in the lignin metabolism pathway [43]. A study in rice revealed that downregulation of *HCT* gene expression reduced lignin content [44]. Our results revealed that 20 HCT genes were identified, eleven *HCTs* were highly expressed in browning calli, and nine were downregulated. The brown callus cells were lignified, and the expression patterns of the *HCT* genes differed between the non-browning callus and the brown callus at the transcriptional level, indicating that *HCT* plays a key role in lignin synthesis in *Camellia hainanica*.

The first specialized step in flavonoid biosynthesis is the generation of tetrahydroxy chalcone by activated p-coumarol coA, catalyzed by CHS. The high expression of *CHS* in many species (such as grapes, cotton, etc.) can improve the resistance of plants to diseases [45]. In addition, the expression levels of the *CHS* and *CHI* genes are positively correlated with the accumulation of total flavonoids in *Lycium barbarum* [46]. The overexpression of the *CHS* gene can induce the synthesis and accumulation of flavonoids in *Linum usitatissimum* seeds [47]. In this study, a total of seven *CHS* genes with differential expression between T2 vs. T1 were identified, all of which were upregulated at T2. On the one hand, the upregulation of *CHS* and *CHI* may increase flavonoid synthesis in *Camellia hainanica* browning calli, providing a basis for synthesizing and oxidizing phenolic substances. On the other hand, the browning of *Camellia hainanica* calli may activate related pathways, which is conducive to resisting the influence of callus browning on cells. In this study, one *4CL* gene and five *F3H* genes were significantly overexpressed in brown calli. Both the *Ps4CL* and *PsF3H* genes are highly expressed in the petiole callus of severely browned peony [48]. The expression of 4CL and PAL in apple, kiwi, loquat, pear, and other fruits was confirmed to be proportional to lignin synthesis [2,49]. Combined with the results of this study, it is speculated that *4CL* and *F3H* may participate in and regulate the browning of *Camellia hainanica* calli. DFR is a key enzyme that catalyzes the stereospecific reduction of dihydroflavonols to anthocyanins. Under stress conditions, the overexpression of *DFR* in sweet potato enhanced the scavenging of reactive oxygen species in plants [50]. The DFR substrate, dihydroflavonols, can be catalyzed by FLS to produce flavonols, while the white anthocyanins produced by DFR can then be converted to proanthocyanidins by LAR [51]. Our RNA-Seq results identified that one *DFR* gene was strongly and highly expressed in T2, which may be because browning activated the oxidative stress response of *Camellia hainanica* callus to resist the influence of browning on the development of *Camellia hainanica* callus. However, *FLS* had different expression patterns in T1 and T2 callus; that is, three *FLS* were highly expressed in T2, shunting the substrate of DFR to some extent. Metabolomics also indirectly confirmed our conjecture. Metabolites in the T2 vs. T1 group identified a large number of differentially accumulated oxides, while no browning-related oxides such as POD and PPO were detected. In addition, differentially expressed LAR, ANS and ANR genes were identified in T2 vs. T1, which catalyzed the products catalyzed by DFR into different PAs.

All the genes encoding these enzymes are highly enriched in the callus of Camellia hainanensis, which may lead to the accumulation of phenolic substances and lignin. Metabolomic results revealed that the content of phenolic acid metabolites (catechins, epicatechins, and chlorogenic acid) in browning tissue was reduced, which is consistent with the findings of a study in Bayberry [52].

### 3.4. TFs Involved in the Browning of Camellia hainanica Calli

Transcription factors of MYB, bHLH, and WRKY all regulate growth, development, and secondary metabolism and are the most important transcription factor families in plants [53]. The three TFs directly or indirectly regulate other branches of the phenylpropane pathway, affecting the synthesis of compounds such as flavonoids, anthocyanins, or lignin [54]. In this study, thirty *MYB* genes, twenty-seven *bHLH* genes, and twenty-six *WRKY* genes were identified with different express genes in the RNA-Seq samples in the T2 vs. T1 group. In other reported studies, these genes have been shown to be involved in regulating flavonoid biosynthesis. *PpMYB* family members activate *PpCHS*, *PpCHI*, *PpF3H*, *PpFLS*, and other genes in the flavonoid synthesis pathway of pear fruit, regulating flavonol biosynthesis in fruit [55]. In sea buckthorn fruit, members of the *HrbHLH* family regulate the content of flavonoids through interactions with *PAL* and *CHS* [42]. *WRKYs* are involved in regulating the browning process of fresh-cut apples [56]. Therefore, MYB, bHLH, and WRKY transcription factors may be involved in the browning process of *Camellia hainanica* callus. However, their functional properties require further exploration to elucidate their potential effects on the browning of *Camellia hainanica* calli.

In this study, we used physiological, widely targeted metabolomics and transcriptomic methods to reveal the cellular changes that occur during callus browning and investigated the effects of callus browning on transcriptional metabolism at the metabolite and gene levels. Our results revealed an obvious accumulation of polyphenols and lignified substances in the calli of *Camellia hainanica* at different stages of callus browning. In summary, the browning of the callus of *Camellia hainanica* is mainly caused by enzymatic browning. The accumulation and oxidation of flavonoids are crucial and decisive steps in this process. We find that Candidate genes (*PAL*, *4CH*, *F3H*, *MYB*, *bHLH*, etc.) related to the accumulation of phenols were identified during the browning of the callus of *Camellia hainanica*. However, further research is needed to determine whether these candidate genes are directly involved in flavonoid biosynthesis in *Camellia hainanica* and how they affect the accumulation of phenolic substances and callus browning. Future studies should focus on functional validation of these candidate genes using CRISPR-Cas9 (Clustered Regularly Interspaced Short Palindromic Repeats) technology and study their direct role in flavonoid biosynthesis and callus browning decision, thus laying the foundation for screening ideal explants inducing calli and improving callus survival.

## 4. Materials and Methods

### 4.1. Plant Material

The plant materials used in the experiment were *Camellia hainanica* “Reyan No. 2” oil tea with high yield and excellent characteristics, which were planted at the base of the Coconut Research Institute of the Chinese Academy of Tropical Agricultural Sciences (Wenchang, China). The immature leaves of “Reyan No. 2” were used as explants to induce calli. When the explants were prepared, they were disinfected with 70% ethanol and 84 disinfectant for 60 s and 15 min, respectively, and then washed with sterile water 5 times for 5 min each. The main leaf veins and edges were then carefully removed, and the leaf explants were cut into approximately 0.5 mm squares, placed on Murashig and Skoog (MS) medium (PhytoTechnology, Lenexa, KS, USA) supplemented with 2 mg/L 2,4-D + 2 mg/L 6-BA, and cultured in the dark at 25 °C for 16–8 h to induce and culture the calli. The severity of callus browning can be divided into three stages: the T1 stage at 2 weeks, when the callus is induced into milky white; the T2 stage, when the callus is 6~8 weeks old and light brown; and the T3 stage, when the callus appears dark brown at 14–16 weeks of age. The plant hormone products used above are purchased from Beijing Solarbio Science and Technology Co., Ltd. (Beijing, China).There were 6 replicates for broad target metabolomics and RNA-Seq, and the collected tissue samples were immediately frozen in liquid nitrogen, wrapped in aluminum foil, and stored at −80 °C until further analysis.

### 4.2. Histochemical Observation of Callus Browning

The fresh callus was immediately placed in the tissue-fixing solution at room temperature for 24 h and placed in the embedding frame. The samples were dehydrated with different alcohol (Sinopharm Chemical Reagent Co., Ltd., Shanghai, China) concentrations, cleared with xylene (Sinopharm Chemical Reagent Co., Ltd., Shanghai, China), and immersed in paraffin at 65 °C. The wax-impregnated tissue was embedded in the embedding machine and cooled in a −20 °C freezer. A paraffin slicer was used to cut the samples into 4 μm thick slices, which were baked at 60 °C, removed at room temperature, and stored for later use. The sections stained with Ferrante solid green were stained with the corresponding dyeing solution for 2 min, and the excess dye was washed off with tap water and decolorized with 50~80% alcohol. After 5–10 min with aniline blue staining solution, the slices were washed with tap water and shaken in an oven at 60 °C. Both stained sections were sliced with clean xylene for 5 min and sealed with neutral gum. Microscopic (Nikon Eclipse, Tokyo, Japan) inspection, image acquisition, and analysis were performed. After safranin (Servicebio, Wuhan, China) and O-fast green staining (Servicebio, Wuhan, China), the cellulose cell walls were stained green, whereas the lignified cell walls appeared red. The walls of viable cells displayed a reddish-purple color after toluidine blue staining, whereas dead cells without cytoplasm presented a blue to greenish color.

### 4.3. Metabolite Extraction and Profiling

The T1, T2, and T3 oil tea callus samples were thawed on ice. Each 100 mg sample was extracted with 1 mL of precooled 50% methanol buffer, swirled for 1 min, and cultured at room temperature for 10 min. The extract was kept at −20 °C overnight. After centrifugation at 4000× *g* for 20 min, the supernatant was transferred to a new 96-well plate. In addition, 10 μL of each extraction solution was taken to prepare mixed QC samples. All samples were collected via the LC-MS system according to the manufacturer’s instructions. The UltiMate 3000 UPLC system (Thermo Fisher Scientific, Bremen, Germany) and an ACQUITY UPLC T3 column (100 mm × 2.1 mm, 1.8 µm, Waters, Milford, CT, USA) were used for reversed-phase separation. The column oven was maintained at 40 °C. The mobile phase comprised 0.1% formic acid in water (A) and solvent acetonitrile (B). The low rate was 0.3 mL/min, and the mobile phase consisted of solvent A. Gradientelution conditions were set as follows: 0~0.8 min, 2% B; 0.8~2.8 min, 2% to 70% B; 2.8~5.6 min, 70% to 90% B; 5.6~6.4 min, 90% to 100% B; 6.4~8.0 min, 100% B; 8.0~8.1 min, 100% to 2% B; 8.1~10 min, 2% B.

Metabolites eluted from the column were detected using a high-resolution tandem mass spectrometer, TripleTOF 6600 (SCIEX, Framingham, MA, USA). The Q-Exactive was operated in both positive and negative ion modes. The ion spray floating voltage was set to 5000 V for the positive ion mode. The ion spray floating voltage for the negative ion mode was set to −4500 V. Data acquisition was performed in data-dependent acquisition (DDA) mode. The mass range of TOF is 60~1200 Da. Precursor spectra (70–1050 *m*/*z*) were collected at 70,000 resolution to hit an AGC target of 3 × 10^6^. To assess the stability of the LC-MS during the whole acquisition, a quality control sample (pool of all samples) was acquired after every 10 samples.

### 4.4. Metabolomics Data Analysis

XCMS software (Version: 3.4.1, https://www.ncbi.nlm.nih.gov/pubmed/16448051, accessed on 30 June 2024) was used to preprocess the collected mass spectrum data. The original LC-MS data file was converted into mzXML format. Then R software (version 4.0) was used to perform the statistical analysis of the data and the processing of the XCMS, CAMERA, and metal toolboxes. The metabolites were labeled via the online KEGG (Version: 2021.05, http://www.genome.jp/kegg/, accessed on 3 July 2024) and Human Metabolome Database (HMDB, https://hmdb.ca/, accessed on 3 July 2024), and the samples’ precise molecular mass data (*m*/*z*) were matched with the data in the database. An in-house database was used to verify the metabolite identification.

### 4.5. RNA-Seq and Data Processing

The mRNA of each sample was isolated and purified with TRIzol reagent (Thermo Fisher, Guangzhou, China). The amount and purity of total RNA were subsequently controlled with a NanoDrop ND-1000 (NanoDrop, Wilmington, DE, USA), and its integrity was tested with a Bioanalyzer 2100 (Agilent, Santa Clara, CA, USA). The concentration was >50 ng/μL, the RIN value was >7.0, and the total RNA concentration was >1 μg. The mRNA with PolyA (polyadenylate) was specifically captured by two rounds of purification using oligo (dT) (Dynabeads Oligo (dT), cat.25–61005, Thermo Fisher, Guangzhou, China) beads. The captured mRNA was segmented by magnesium ion interruption kit (NEBNextR Magnesium RNA Fragmentation Module, cat.E6150S, Ipswich, MA, USA) at 94 °C for 5–7 min. The fragment was reverse transcribed to synthesize cDNA. The obtained fragment sizes were screened and purified via a series of methods, followed by PCR to form a 300 bp ± 50 bp fragment size library. Finally, we used Illumina NovaSeq^TM^6000 (LCBioTechnology Co., Ltd., Hangzhou, China) to double-end sequencing in PE150 mode according to standard procedures.. After the sequencing data were obtained, they were filtered first to obtain high-quality sequencing data (clean data).

### 4.6. Transcriptomic Data Analysis

Transcription essentiality control, assembly, and quantification were performed with FastQC (version: 0.10.1) and StringTie (version: 2.1.6), respectively. Then, HISAT2 (version 2.2.1) was used to compare the high-quality sequencing data to the camelia reference genome [57]. DEGs were characterized via DESeq2/edgeR (version 1.22.2/3.22.5), with an error finding rate threshold < 0.05 and logarithmic 2-fold change absolute value ≥1 standard. Annotation of DEGs was performed via BLAST searches of their cDNA or protein sequences against the Swiss-Prot (http://www.expasy.ch/sprot/, accessed on 13 July 2024) and UniPro protein databases (https://www.uniprot.org/, accessed on 16 July 2024), followed by enrichment analysis of KEGG pathways (Version: 2021.05, http://www.genome.jp/kegg/, accessed on 19 July 2024) and GO terms (Version: 2021.05, http://www.geneontology.org, accessed on 19 July 2024). RNA-seq and metabolite assays were performed with the assistance of LC-Bio Technologies (Hangzhou) Co., Ltd. (Hangzhou, China).

## 5. Conclusions

Callus browning is a common phenomenon in the tissue culture of *Camellia hainanica*. After a period of aseptic culture, callus browning generally occurs before somatic embryo induction, leading to severe cell death. Histological staining revealed an obvious accumulation of polyphenols and lignified substances in the calli of *Camellia hainanica* at different stages of callus browning. That is, with the occurrence of browning in the callus of *Camellia hainanica*, the lignin in the callus increased, and polyphenols accumulated in the cells. Comprehensive transcriptome and metabolomics analyses revealed that during callus browning, the expression of genes regulating flavonoid biosynthesis was significantly up-regulated, and the synthesized intermediates provided the basic substances for the synthesis of lignin, tannins, and phenols. The identified metabolites enhanced, such as catechins and epicatechins, also confirmed the above results. Hence, during callus induction, *Camellia hainanica* tissue with fewer phenolic substances can be selected as explants; in the culture of explants and callus, adding adsorbents and antioxidants reduces the oxidation of phenolic substances. In addition, proper temperature reduction can reduce the enzyme activity in the *Camellia hainanica* callus, which is conducive to alleviating browning. Overall, this study reveals the molecular mechanism of callus browning in *Camellia hainanica*, which laid a foundation for improving the efficiency of tissue culture and the genetic transformation of *Camellia hainanica*. However, the specific functions of the candidate genes identified in this study require further investigation.

## Figures and Tables

**Table 1:** Summary of the RNA-Seq data.

Sample	Valid Reads	Raw Bases	Mapped Reads	Q20%	Q30%	GC%
T1_1	33,823,714	5.23 GB	27,764,079 (82.08%)	97.65	92.78	44.17
T1_2	34,812,368	5.36 GB	28,870,807 (82.93%)	97.74	92.99	44.06
T1_3	33,808,954	5.20 GB	27,875,212 (82.45%)	97.79	93.15	43.76
T1_4	38,377,496	5.92 GB	32,096,526 (83.63%)	97.89	93.52	44.46
T1_5	39,392,152	6.08 GB	33,228,806 (84.35%)	97.76	93.16	43.68
T1_6	40,398,604	6.22 GB	34,003,340 (84.17%)	97.90	93.56	44.18
T2_1	37,534,600	5.80 GB	31,329,260 (83.47%)	97.84	93.38	44.63
T2_2	39,768,790	6.14 GB	33,400,304 (83.99%)	97.77	93.16	44.54
T2_3	39,895,370	6.16 GB	33,600,184 (84.22%)	97.80	93.28	44.68
T2_4	35,123,964	5.42 GB	29,794,296 (84.83%)	97.90	93.58	44.89
T2_5	33,238,864	5.13 GB	28,025,033 (84.31%)	97.88	93.55	44.51
T2_6	37,886,492	5.88 GB	31,389,973 (82.85%)	97.83	93.32	44.67
T3_1	39,586,342	6.11 GB	32,963,243 (83.27%)	97.87	93.46	44.51
T3_2	37,899,240	5.85 GB	31,642,750 (83.49%)	97.85	93.42	44.58
T3_3	39,301,424	6.07 GB	32,853,719 (83.59%)	97.85	93.39	44.82
T3_4	33,443,562	5.16 GB	28,187,993 (84.29%)	97.91	93.62	44.91
T3_5	39,603,272	6.13 GB	33,307,938 (84.10%)	97.81	93.3	44.88
T3_6	34,536,542	5.34 GB	29,053,042 (84.12%)	97.91	93.63	44.83

Note: Sample: The sample name. Valid Reads: The number of valid reads. Valid Bases: The amount of valid data; Mapped reads: The number of reads that can be matched to the genome. Q20%: The proportion of bases with a mass value ≥20 (sequencing error rate less than 0.01). Q30%: The proportion of bases with a mass value ≥30 (sequencing error rate less than 0.001). GC%: The proportion of GC content.

## Data Availability

The data presented in the study were deposited in the National Center for Biotechnology Information (Accession Number: PRJNA1157213).
